# Tell Your Story: Strengthening Scientific Engagement and Welfare Leadership among Animal Technologists

**DOI:** 10.12688/wellcomeopenres.25958.3

**Published:** 2026-08-10

**Authors:** Sian Murphy, Christophe Galichet, James Baker, Luke O'Hara, Jordi L-Tremoleda, Eleni M Amaniti

**Affiliations:** 1 Neurobiological Research Facility, Sainsbury Wellcome Centre, University College London, London, W1T 4JG, UK; 2 Blizard Institute/Biological Services, Queen Mary University of London, London, UK

**Keywords:** Animal technologists, Training, Workshop, Poster, Welfare

## Abstract

**Background:**

Animal Technologists (ATs) are vital in the scientific community, offering crucial expertise in animal care, welfare, and experimental procedures. As projects become more complex, there is an increasing opportunity for ATs to engage actively in scientific projects, decision-making, and collaborative research processes. Unfortunately, many ATs may feel that their professional background is insufficient or may not consistently recognise the significant scientific value of their daily activities. These perceptions can result in missed opportunities for personal and professional growth, and for advancing Refinement and Reduction initiatives within and outside their establishments.

**Methods:**

In developing the resource, our pilot study took into account commonly reported barriers to ATs engagement in scientific communication and collaboration, including perceptions of role boundaries and confidence in interactions with researchers and management. We describe the development of a practical resource designed to support ATs’ self-awareness, confidence, and engagement in scientific discussions by helping them articulate their expertise clearly and effectively. The resources were evaluated during “Tell Your Story” workshops.

**Results:**

Feedback from the workshops was consistently positive. Participants reported increased confidence in recognising and communicating the scientific value of their work, as well as improved engagement in scientific discussions and collaboration.

**Conclusions:**

By combining an understanding of the challenges with practical tools and clear strategies, this pilot study suggests that ATs can be supported to recognise and develop their roles as knowledgeable scientific contributors and welfare advocates. This enables ATs to confidently share their expertise, advance their careers, enhance research quality and reproducibility, support the 3Rs principles, and strengthen the culture of care within a more collaborative and inclusive scientific research environment.

## Plain Language summary

Animal Technologists (ATs) play a vital role in science through their expertise in animal care, welfare, and experimental delivery. However, many ATs do not always feel confident taking part in scientific discussions or recognising the scientific value of their everyday work. This can lead to missed opportunities to improve animal welfare, reduce animal use, and strengthen research quality.

This work explains the benefits of ATs becoming more actively engaged in scientific conversations and collaborative research environments and identifies common barriers they face when working with researchers and management. It also explores practical ways to improve communication, confidence, and inclusion. To support ATs, we developed a practical resource designed to help them recognise and communicate the scientific value of their contributions more clearly. These resources were evaluated during “Tell Your Story” workshops, and participant feedback was very positive.

Overall, by recognising ATs as key contributors to scientific quality and animal welfare and providing practical tools to support their engagement, ATs can feel more confident, develop their careers, contribute to better science, support the 3Rs principles, and help strengthen a collaborative and inclusive culture of care within research environments.

## Introduction

Animal Technologists (ATs) play a vital, yet often undervalued, role in biomedical research. They possess expertise in animal welfare, husbandry, and experimental delivery and support, which directly influences research quality. However, the contributions of ATs to scientific outcomes and interdisciplinary collaboration remain limited, underexplored, and often undervalued.

Qualitative evidence suggests that ATs actively support the 3Rs principles (Replacement, Reduction, and Refinement). For example, an ethnographic study of UK university facilities showed that ATs are inventive and perceptive in enhancing animal welfare, care, and use. The authors concluded that this aspect of the ATs’ role had not yet been officially recognised. ^1^ Furthermore, in Spain, a survey involving 356 participants in biomedical research, including technicians and animal care staff, found that many technicians rarely discussed their work beyond their immediate inner circles. ^2^ Similarly, an international survey found that ATs value sharing 3Rs-related ideas but feel less confident as scientific contributors, highlighting a mismatch between technical expertise and perceived scientific involvement. ^3^

**Figure 2.**
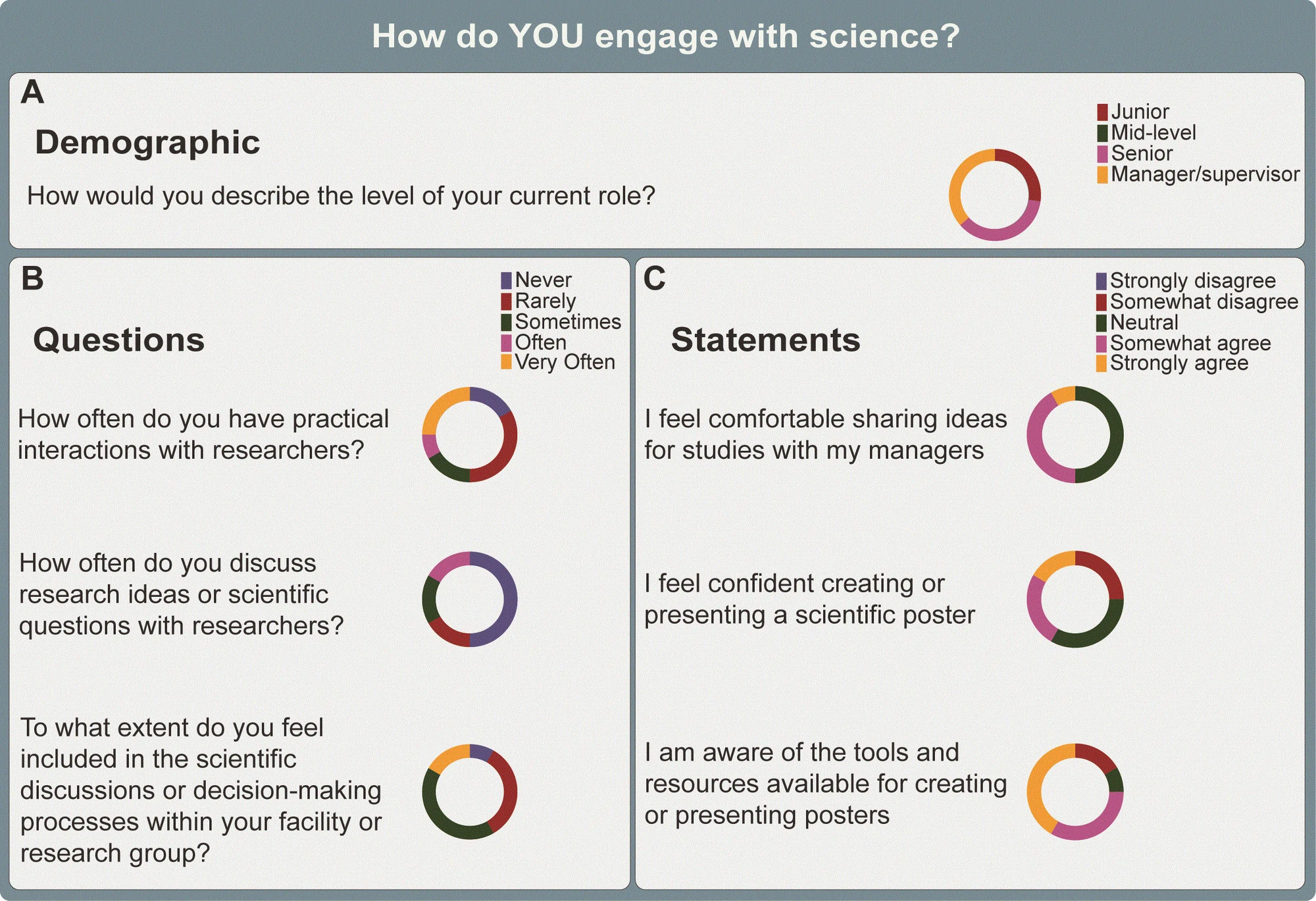
“ *How do YOU engage with science?*” survey results. Pie charts showing the results of the “How do YOU engage with science?” survey including respondent demographics (A), their responses to the questions (B), and their responses to the statements (C). Chi-square goodness-of-fit test values and p values are 2.167, 0.7051; 8, 0.091 and 7.167, 0.1273 for questions (B), top to bottom, and 13.83, 0.0078; 3.83, 0.4290 and 7.167, 0.1273 for statements (C), top to bottom. Degrees of freedom = 4; n = 12 for all statistical tests.

**Figure 3.**
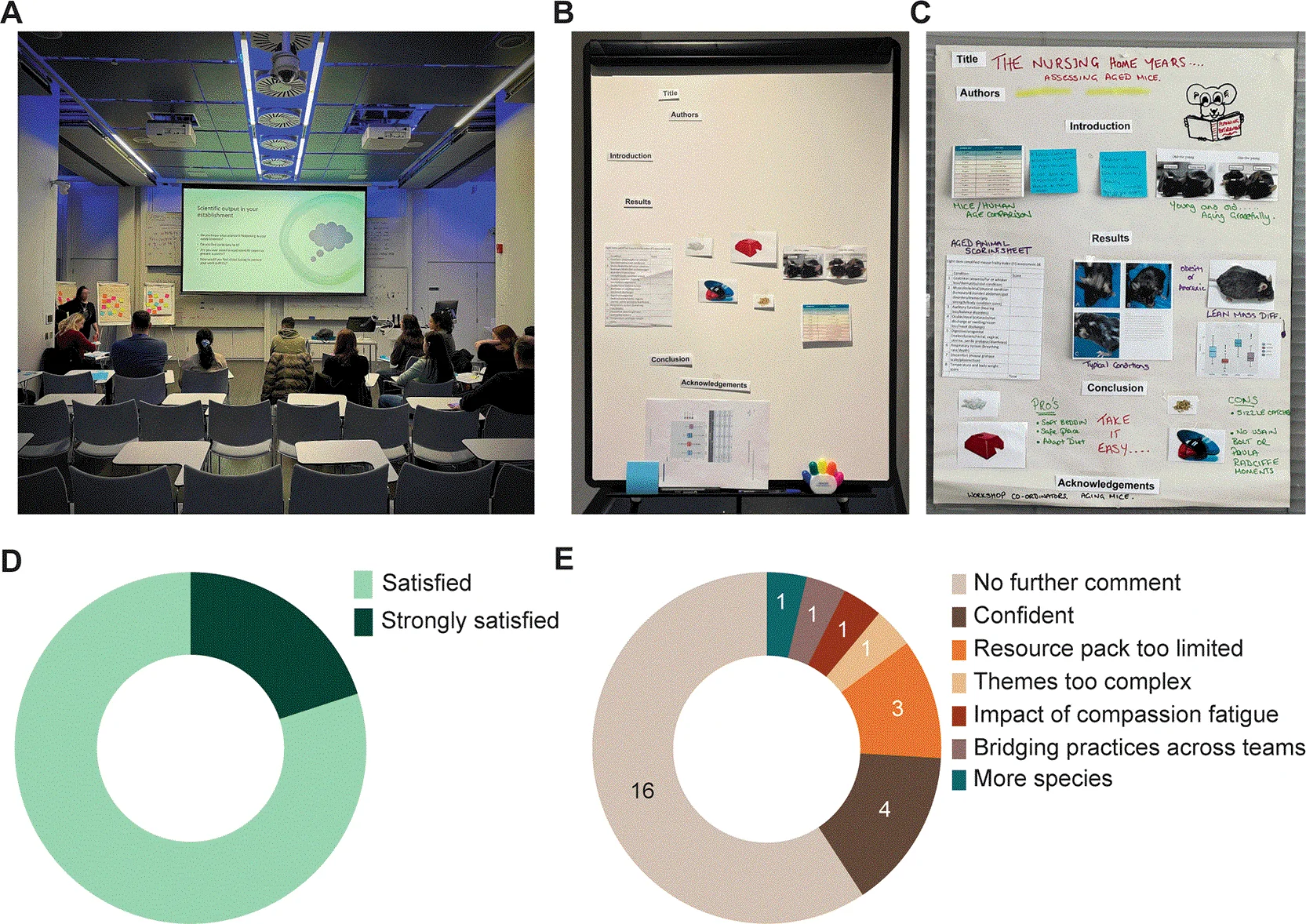
Workshop activities and outcomes. (A) Photo taken during activity 1 (talk) at the workshop held at the SWC, showing the collaborative setting. (B-C) Photos showing the poster design at the beginning of activity 2, when the resource pack is random (B), and after the poster design has been completed (C). (D-E) Pie charts showing the participants’ satisfaction levels at the end of the workshop (D) and their comments (E), which help refine subsequent workshops.

Bridging this gap is crucial for enhancing ATs’ skills, ensuring their full integration into institutional and research roles, and successfully implementing the 3Rs. When ATs’ scientific input is overlooked, their everyday observations and practical knowledge are often excluded from discussions that focus on refinement, welfare issues, and research advancements. This may mean early signs of negative welfare, procedural difficulties, or excessive breeding surplus are not fully recognised or acted upon. As a result, opportunities to improve animal welfare, refine procedures, reduce unnecessary animal use, and strengthen research quality may be delayed or lost. Empowering ATs to communicate effectively and participate in scientific discussions and decision-making processes is, therefore, central to strengthening the 3Rs and fostering a more inclusive institutional Culture of Care. This perspective aligns with broader Culture of Care initiatives that emphasise staff engagement, reflection, and shared responsibility within animal research establishments, as highlighted in international collaborative frameworks such as the COST Action IMPROVE. ^4^

In the current pilot study, we present findings on how ATs perceive their engagement with science, identify common communication challenges across research environments, and highlight areas for improving confidence, inclusivity, and professional recognition. These findings point to organisational and cultural barriers that limit ATs’ participation in scientific discussion, despite their direct influence on animal welfare, experimental support, and research quality.

To address these issues, we developed and tested a practical strategy to support ATs engagement through communication and confidence-building via poster making-based workshops (hereafter, “Tell Your Story” workshops). The approach combined short, targeted input on communication in scientific methodology and teaching theories with structured, collaborative poster design activities that positioned ATs as active scientific contributors. Practical tools included basic communication frameworks, team planning, poster design principles, presentation skills, and peer-feedback exercises. The strategy was tested through pilot workshops delivered at two UK research institutions. Effectiveness was assessed using reflective exercises and structured participant feedback, allowing evaluation of perceived confidence, inclusivity, and relevance to professional practice. We finally report the outcomes from these workshops and assess how this approach supports ATs engagement, knowledge sharing, and a collaborative research culture.

By combining a clear understanding of professional and organisational challenges with practical tools and strategies, we aim to show how ATs can be empowered as knowledge-holders, scientific contributors and methodology innovators. This supports research integrity, greater implementation of the 3Rs, and fosters a collaborative and inclusive scientific community.

## Methods, resources and tools

Scientific posters are a visual way to present research or scientific information clearly and concisely, often displayed at conferences or meetings. They combine brief text with visual aids to describe a question, methods, key findings, and conclusions, helping to communicate essential information clearly, quickly and yet impactfully. Posters often help initiate discussion between the presenter and the audience. “Tell Your Story” workshops were developed and delivered to help ATs enhance their scientific communication and scientific poster design skills.

The workshops consisted of a series of slide-based presentations to provide an introductory background on communication, its importance and the different ways to communicate effectively depending on the audience’s perceived priorities; on openness in Laboratory Animal Science welfare and research, which highlighted the importance of effective communication engagement within the research community and; on poster design. The presentations were complemented by a group-based practical activity focused on the design and presentation of scientific posters.

### Participants and procedure

For these pilot workshops, the four participating institutions were identified in advance by the authors using a purposive approach, based on prior expressions of interest in research delivery and the practical feasibility of delivering the workshops within the available time and resources. An invitation outlining the workshop’s aims and objectives was circulated via email to senior management in animal facility departments at these institutions. Facilities that responded positively were included. Institution/facility management was asked to nominate participants who would provide a mix of roles, career stages and experience levels, with self-nominations also considered where appropriate. The final group was selected to ensure a manageable workshop size while maintaining this diversity. Selection was based on expressed interest in scientific communication and poster development, the workshop’s relevance to participants’ roles, and participants’ potential to apply and share the learning within their local research environments. Participants were not selected on the basis of perceived skill deficits. As this was a pilot study, the sample was not intended to be representative of the wider animal research community; however, potential selection bias was considered by seeking participation across different roles, levels of experience and institutional contexts, and this limitation is acknowledged in the interpretation of the findings.

### Surveys

A preliminary survey was developed to ensure that the workshop’s learning outcomes aligned with participants’ needs. A voluntary, anonymous, preliminary survey using a 5-point Likert-scale was conducted prior to the start of the workshop. It included questions on the frequency of participants’ practical interactions with researchers (never, rarely, sometimes, often, very often), their perceived inclusion in scientific discussions and decision-making, and their confidence in creating and presenting a scientific poster (strongly disagree, somewhat disagree, neutral, somewhat agree, agree). Participants also provided information about their professional level (junior AT, mid-level AT, senior AT, or manager). Survey responses were collected in two different formats across the two workshops. In one workshop, responses were collected electronically via Microsoft Forms alongside facilitated discussion, while in the other, responses were collected using Post-it notes on poster easels, again accompanied by group discussion.

At the end of each workshop, the group feedback discussion was complemented by a 5-point Likert-scale feedback form that included a free-text section. The feedback survey focused on participants’ satisfaction with the workshop, the quality of the presentations and the effectiveness of the poster design (strongly not satisfied, not satisfied, neutral, satisfied, strongly satisfied).

### Step-by-step guide

Using elements of the workshop presentation on poster design, an educational poster/handout detailing the essential elements of scientific poster development was created and provided as a resource. “Tell Your Story” poster/handout features a clear design layout and serves as a user-friendly, step-by-step guide (Data availability; extended data: Supplementary Figure 1 and Supplementary Figure 2). ^5^

### Location requirements

Workshops were held at the Sainsbury Wellcome Centre and the University of Oxford. The venue and equipment requirements needed to support the interactive activities are summarised below ( Table 1).

**Table 1.** Workshop logistics and physical requirements.

Category	Specification	Purpose/rationale
Workshop duration	~3 hours (including breaks)	Allows delivery of presentations, practical poster activity, networking, discussion, and feedback
Venue	One spacious room accommodating 12–15 participants, facilitators, and poster easels	Supports group work, movement between activities, and poster presentations
Seating and work surfaces	One chair and table/desk per participant; additional tables for group work	Required for collaborative poster design and note-taking during practical activities
Presentation equipment	1 laptop (facilitator), projection screen, projector	Used for delivery of introductory talks and instructions
Audio equipment	Microphone and speaker (only if room size requires)	Ensures audibility during presentations and group feedback
Poster materials	Poster easels (1 per group), printed templates, stationery (markers, Post-it notes, paper)	Enables hands-on poster design activity
Refreshments	Tea/coffee and water for all participants; light refreshments	Provided during scheduled breaks to support engagement
Breaks	10–15 mins	Scheduled midway through the workshop

### Poster design resources

To facilitate ATs’ engagement during the poster-based workshop (practical activity), we organised the posters by theme. In workshop 1, the poster topics focused on the care and welfare of laboratory animals ( Table 2). Based on feedback from workshop 1, workshop 2 also included topics on roles and career development paths for ATs ( Table 2). Participants were given resource packs containing physical resources ( Table 3), along with supporting materials such as images, research papers, and data sets related to their chosen topics. The average cost of these materials was £75, highlighting the workshop’s low cost. Materials were selected to support rapid prototyping of scientific posters and collaborative design, rather than for aesthetic or artistic purposes.

**Table 2.** Themes & topics used for the poster-based workshop activity.

Theme	Topic	Workshop	Example of sources
Animal Welfare	Aged animal care	2	Characteristics of early and late adult C57BL/6NCrl mice (Charles River)
			Aged C57BL/6 J mice for research studies (The Jackson Laboratory)
	Environmental enrichment	1	Environmental enrichment to reduce aggression in CD-1 male mice ^11^
	Food and fluid control	2	Refinements to rodent fluid/food control (NC3Rs)
			Daily torpor in mammals (Wikipedia)
	New ointment for wound healing	1	Evaluation of wound healing (Derma Wound Healing Ointment)
	Refine ear notching	1	Tattooing ears (AIMS)
			Ear tagging (The Jackson Laboratory)
	Positive welfare	2	Definition of positive animal welfare (The Royal Society)
		2	Playrooms for rats (NC3Rs)
	Post surgery analgesia administration	1	Gel delivery system (MediGel ^®^ Sucralose)
ATs path & career	Career track	2	Animal technologists career path (Institute of Animal Technology)
			A caring career (Institute of Animal Technology)
	Culture of Care	2	Culture of Care in academia (Understanding Animal Research)

**Table 3.** Materials provided for the poster-based workshop activity.

Category	Materials	Purpose
Poster substrate	A2 poster sheets	Used as the primary medium for designing and presenting scientific posters
Writing and annotation tools	Pens, highlighters, coloured markers	Used to annotate, label, and visually structure poster content
Assembly materials	Reusable adhesive, tape	Used to attach printed materials and visual elements to posters
Planning and organisation tools	Sticky notes	Used for outlining poster structure, sequencing content, and group planning
Display equipment	Poster easels	Used to support poster design and facilitate group presentations
Resource packs	Printed templates, example figures, diagrams, and supporting materials	Provided to guide poster layout, content selection, and communication structure

### Software & statistics

The online survey was developed with Microsoft Forms (Microsoft 365). Survey results were assessed by calculating the percentage of respondents for each Likert-scale point and visually presented as parts of a whole using Prism (GraphPad). A Chi-square goodness-of-fit test was used to assess whether responses were evenly spread across the Likert-scale options or whether participants tended to select particular options more often. Data from the 5-point Likert-scale survey were analysed descriptively and, given the pilot nature of the study, explored statistically using an observed-versus-expected approach. As no prior data were available to define an expected response pattern, we used an equiprobable distribution across the five response categories as a simple null benchmark, representing the absence of a systematic response tendency. Proportions and confidence intervals were calculated in Prism (GraphPad) using the Wilson/Brown method, and a chi-square goodness-of-fit test was used to compare observed and expected response distributions. Results were interpreted cautiously and in the context of the limited scope of the pilot study. (Data availability; underlying data). ^5^

The “Tell your story” resources were created with InDesign and Illustrator (Adobe). The figures in this article were generated using Photoshop and Illustrator (Adobe).

## Workshop overview and structure

Two pilot “Tell Your Story” workshops were held, one at SWC and the other at the University of Oxford, involving a total of 27 ATs from four different Higher Education Institutions. Participants ranged from junior staff to managers, including ATs, support personnel, and facility managers, all of whom shape communication in research settings.

The workshops were intentionally structured to promote collaboration, confidence, and peer learning by combining short instructional talks with group-based poster design, peer presentation, and facilitated feedback. This sequence supported progressive learning, reflection, and practical application ( Figure 1).

**Figure 1.**
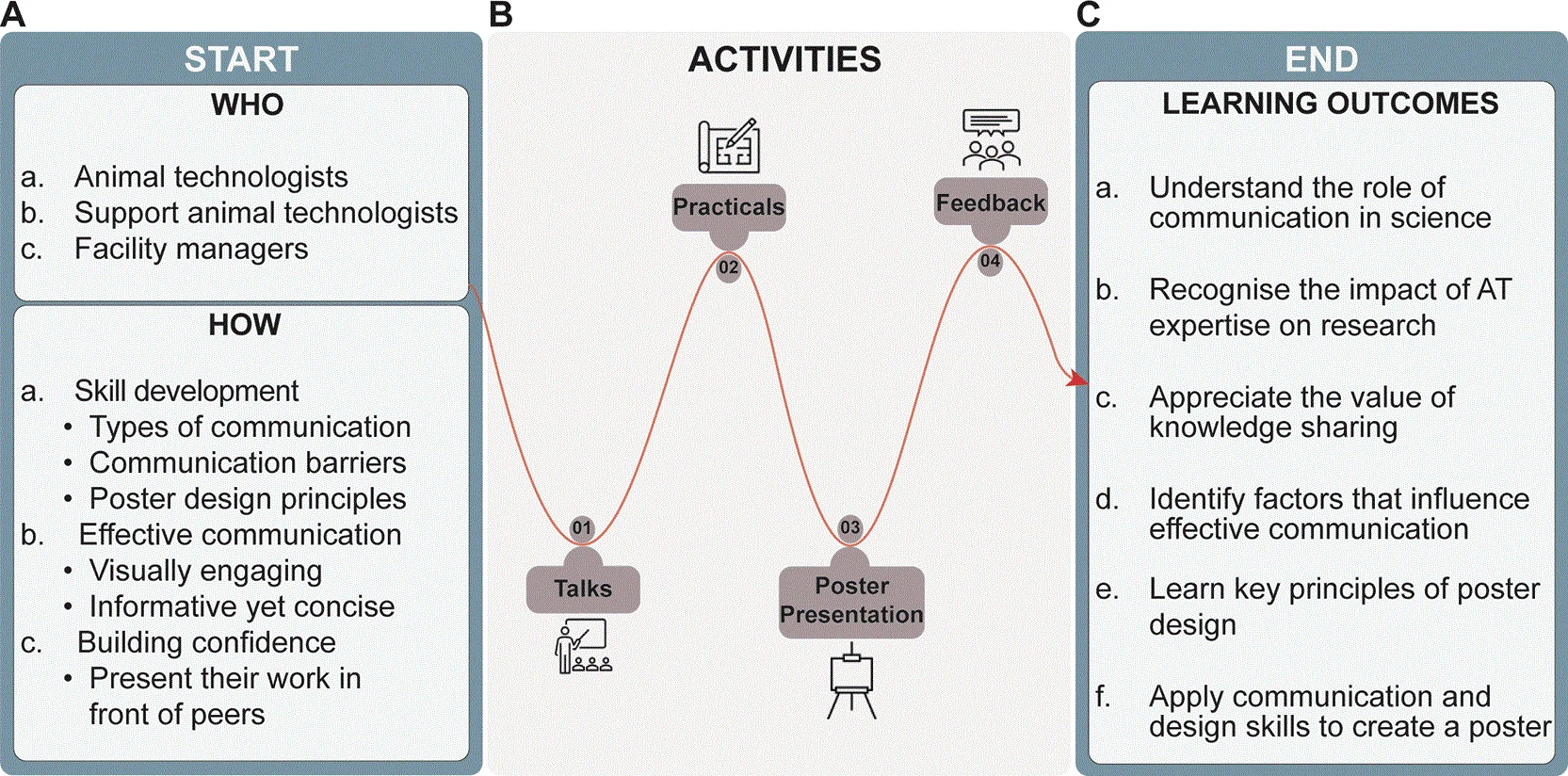
Workshop structure. The workshops were structured around the participants’ needs with three interconnected primary objectives (A). The core of the workshops comprised four activities (B), including talks, poster design, presentations, and feedback. By the end of the workshops, participants achieved a set of learning outcomes (C).

Initially, participants were presented with the workshop’s primary objectives ( Figure 1A): enhancing communication skills, understanding how ATs’ expertise contributes to research, and increasing confidence in presenting scientific concepts. The workshop centred on three interconnected areas of learning:

• **Skill development**, which involved exploring various types of communication, identifying common barriers, and learning poster design basics
• **Effective communication** highlighting strategies for delivering messages that are visually appealing, informative, and concise
• **Building confidence,** which was fostered by encouraging participants to present their work to peers and receive constructive feedback

Four activities formed the core of the programme ( Figure 1B). These included:

• **Talks**, offering an overview of communication in science, its influence on research integrity, the scientific importance of AT expertise, and the universal structure of designing scientific posters
• **Practical activity**, where participants collaboratively worked on preparing real-life posters
• **Poster presentation**, participants presented their posters and gained useful feedback from peers and facilitators during this activity
• **Group feedback and discussion**, during which participants reflected on the workshop, what they had learned, how to apply these skills at work, and gave their feedback

The participants gained from the workshop a set of learning outcomes which comprised ( Figure 1C):

• Understand the role of communication in science
• Recognise the impact of AT expertise on research
• Appreciate the value of knowledge sharing
• Identify factors that influence effective communication
• Learn key principles of poster design
• Apply communication and design skills to create a poster

The four main program activities that served as the structure for the workshops were as follows:

### Activity 1: Communication in science

The first activity began with brief, targeted oral presentations highlighting how effective communication supports scientific clarity, engagement, and transparency, and then explored how this leads to scientific quality, reproducibility, and integrity. Participants learned that communication is not just a supporting skill but a core part of rigorous scientific work, affecting data interpretation, welfare decisions, and knowledge sharing across teams.

To promote self-reflection, participants completed a 5-point Likert-scale questionnaire titled “ *How do YOU engage with science?*” ( Figure 2). The preliminary survey, conducted in three formats during the two workshops, electronically via a QR code, via Post-it notes and verbally, aimed to gather quantitative insights into ATs’ levels ( Figure 2A), experiences, motivations, and perceived barriers, while also encouraging reflection on communication within their workplaces. This exercise highlighted several recurring challenges, such as limited cross-laboratory animal science/research team dialogue, inconsistent information flow between technical and research staff, and weak feedback mechanisms that can impede collaboration and ongoing improvement ( Figure 2B-C). The data provided valuable context for understanding how ATs view their role within the broader research environment and highlighted potential gaps in professional development or recognition.

The presenters were senior managers with experience in education, training and poster design. They highlighted how ATs’ technical expertise can influence animal welfare, experimental delivery, data quality and reproducibility. Communication was presented both as a scientific skill, needed to share procedural and welfare information accurately, and as a collaborative skill that supports trust and teamwork ( Figure 3A). Examples showed how transparent communication between technicians and researchers can improve animal care, experimental planning and welfare outcomes. Technicians’ reports of subtle health or behavioural changes—such as weight loss, barbering or altered activity—can inform adjustments to monitoring, procedures, enrichment and handling. Good communication also supports blinding, randomisation and the reduction of confounding factors, leading to more timely interventions and greater experimental consistency.

This emphasised that developing communication skills not only boosts personal confidence and career growth but also elevates the overall quality and integrity of the science conducted. Finally, the presenters highlighted scientific communication through posters and provided participants with general information on poster design and preparation.

Building on these reflections, the second activity put these principles into action with a collaborative, hands-on poster design session. This practical exercise helped participants apply their knowledge of effective communication by translating scientific concepts into clear, visually appealing messages for different audiences.

### Activities 2 and 3: Designing and presenting posters

The second activity put theory into practice through an interactive session designed to reinforce learning and foster creative thinking. Participants were assigned to groups of 3–5 individuals, intentionally composed to include a mix of roles and experience levels, such as junior and senior ATs, support staff, and, where applicable, facility or unit managers. Each group was provided with a themed resource pack based on familiar scenarios, such as improving animal welfare, promoting refinement, or strengthening teamwork, and included templates, stationery, and design tools ( Figure 3B, Table 2 and Table 3). The task was deliberately open-ended, allowing participants to interpret the themes freely and incorporate their personal and workplace experiences. Over 45 minutes, with support from the facilitators (presenters from activity 1), each team designed a poster conveying a key message on topics relevant to ATs ( Table 2). For example, one group presented a poster on aged animal care that highlighted early welfare indicators and refinements to monitoring schedules ( Figure 3C).

This exercise prompted participants to consider practical ways of conveying complex ideas in an engaging and accessible manner. Many teams explored different layouts, colours, and tones to enhance clarity and visual appeal, demonstrating an intuitive understanding of the principles covered earlier in the workshop. The design activity encouraged lively discussions on both balancing scientific accuracy with simplicity and presenting technical information in ways that various audiences, from researchers and technicians to facility inspectors/auditors and the public, could understand.

The poster topics were deliberately selected to reflect scenarios in which ATs may need to communicate with researchers or facility managers regarding experimental practice, welfare observations, or opportunities for refinement. For example, topics such as aged animal care, food and fluid control, post-surgery analgesia, wound healing, environmental enrichment, and ear-notching refinement require ATs to translate practical observations into clear recommendations that may affect monitoring schedules, procedural timing, welfare assessments, or experimental consistency. Groups were therefore encouraged to frame their posters around four practical questions: what has been observed, why it matters for welfare or research quality, what evidence or experience supports the message, and what action or refinement should be considered.

Upon completion of the design activity, each group presented their poster to peers and facilitators, providing an opportunity to practise communicating scientific content and to explain design and content choices. Feedback from peers and facilitators focused on clarity of the scientific message, visual structure, and appropriateness for different audiences, while also identifying specific areas for improvement. This structured peer-feedback process mirrored standard scientific poster activities and reinforced the value of open dialogue and collaborative learning.

Informal observations from facilitators revealed high engagement and collaboration among all groups, with participants expressing enthusiasm, creativity, and pride in their collective work. Overall, these activities improved participants’ understanding of communication as a vital scientific skill, boosted their self-efficacy, and strengthened professional relationships within and across facilities.

### Activity 4: Feedback

To conclude each “Tell Your Story” workshop, participants were asked to complete a brief feedback survey, which included a free-text section for comments. The evaluation focused on participants’ satisfaction with the quality and relevance of the presentations, the perceived knowledge gained, and the effectiveness of the workshop’s practical activity. This feedback helped determine if the workshop met its primary objectives, to enable ATs to feel more confident in communicating their work and to increase their engagement with the broader research community ( Figure 3D-E).

Analysis of the feedback showed high participant satisfaction overall ( Figure 3D). Most respondents felt the workshop achieved its goals, offering valuable insights and practical skills directly useful in their professional roles.

Several constructive suggestions for improvement were received and carefully considered for future workshops. One participant emphasised the importance of tackling compassion fatigue within the AT community. While this topic was outside the workshop’s main learning goals, its significance was recognised, and participants were advised to consult other training resources and support networks dedicated to emotional well-being ( Figure 3E). Four participants noted that the workshop content was slightly below their current expertise, as they already felt confident in designing and presenting posters. This feedback led to the idea of offering differentiated or advanced workshops in the future, enabling participants to engage with material suited to their professional level ( Figure 3E).

Another participant expressed a desire to learn how to bridge communication gaps between researchers and technologists to promote a more integrated, collaborative environment rather than an “us-versus-them” attitude. Additional feedback called for a wider variety of materials in the resource packs, such as journal excerpts, graphs, figures, and supporting literature, as well as broader poster themes to foster creativity ( Figure 3E).

## Discussion

The “Tell Your Story” workshops showed that focused communication and practical skills training can empower ATs to recognise and articulate the scientific aspects of their work. As this was a pilot study, the findings are necessarily limited in scope; however, participants reported that the activities helped them recognise their daily observations and problem-solving as authentic scientific activities that involved curiosity, hypothesis testing, and evidence-based refinement. This change in outlook reinforced the idea that ATs are not just support staff but can apply scientific reasoning and method to improve welfare, refine procedures, and contribute directly to experimental planning, execution, and data quality, rather than solely supporting research activity, by increasing their confidence to document observations, raise welfare-related concerns, and engage more actively in dialogue with researchers and managers.

### ATs as scientific contributors and innovators

ATs contribute to science by identifying welfare issues, testing refinements, or developing better husbandry practices. They are involved in inquiry-driven work that directly influences research results. The workshops offered a structure for recognising and clearly communicating these contributions, specifically through guided reflection, peer discussion, and framing routine observations as data that can inform scientific decision-making. Participants who initially saw little scientific value in their work gradually gained confidence in describing their observations as data and framing their problem-solving as evidence-based innovation, thereby increasing the likelihood that their insights are shared, considered, and acted upon within research teams.

This recognition is more than symbolic - it challenges the conventional divide between “researchers” and “technicians,” emphasising that everyone involved in animal care, monitoring, husbandry, procedural interventions, and *in vivo* follow-up measurements contributes to the collective research process. When ATs receive support to engage in scientific communication, their contributions are recognised and appreciated, resulting in tangible gains for improving the granularity and transparency of animal care, welfare monitoring, and *in vivo* interventions, facilitating earlier identification of welfare concerns, integration of refinements into protocols, and shared decision-making, thereby strengthening the interpretation and reproducibility of experimental data. Such relationships between animal care practices and experimental outcomes have been documented through ethnographic and empirical analyses of AT roles within UK research establishments. ^1^

In parallel, community-driven and cross-institutional initiatives such as the Animal Research Nexus Programme (AnNex) ^6^ have demonstrated the value of structured platforms that support networking, visibility, and knowledge exchange across the wider animal research community, including themes directly relevant to Laboratory Animal Science, welfare, and cultures of communication. ^7^ In addition, the International Culture of Care Network provides a structured forum for sharing ideas and strengthening communication and recognition of contributions within establishments. ^8, 9^ Together, these wider communities of practice complement local, skills-based interventions such as “Tell Your Story” by reinforcing cross-role dialogue, visibility, and shared learning beyond individual facilities.

Such support in communication should not be limited to poster-based outputs. While training in scientific writing and publication was beyond the scope of the “Tell Your Story” workshops, these activities represent an important area for future development. ATs have demonstrated their ability to lead rigorous, welfare-driven research across a range of contexts, including work on nesting material suitability in mice with head plates that supported both animal recovery and experimental consistency, ^10^ and interventions to reduce aggression in CD-1 male mice that minimised injuries and improved the reliability of behavioural outcomes. ^11^ These examples highlight the potential for ATs to contribute more widely to the peer-reviewed literature when appropriately supported. Expanding communication initiatives to include training in scientific writing and publication could help ensure that ATs’ knowledge and experience are more clearly captured in the scientific literature. Over time, this could strengthen welfare-focused evidence, support more consistent research practices, and, in some cases, help reduce unnecessary variation and animal use. Taken together, this highlights the important role that well-considered, species-appropriate welfare practices play in supporting ethical and reliable science.

### Communication as empowerment and integration

Effective communication is essential for bridging institutional and hierarchy gaps. Many ATs voiced frustration over limited dialogue with research staff or management, which restricted their influence on scientific planning and welfare decisions. However, ATs’ ability to contribute to scientific discussions relies on expertise gained through various channels. These include formal professional training, such as Institute of Animal Technology (UK-based) qualifications in laboratory animal science and technology, as well as ongoing professional development, legislation-related training, local competency assessments, and supervised in-house instruction. These routes help ATs develop practical and scientific knowledge in areas such as species-specific behaviour, handling, breeding, health monitoring, pain and distress recognition, procedural support, post-procedural care, record-keeping, humane endpoints, and the principles of the 3Rs. This formal learning is complemented by daily hands-on learning, in which repeated animal observations, participation in husbandry routines, and interactions with researchers, veterinarians, and designated personnel enable ATs to understand how experimental procedures affect animals in practice. Encouragingly, workshops indicated that improving communication channels can better facilitate the sharing and application of this expertise, leading to refinements, improved welfare outcomes, and better scientific planning.

Despite progress, systemic barriers persist. Limited funding, time, and staffing often restrict the scope of ATs’ work and hinder their ability to pursue initiatives beyond immediate operational tasks. In resource-constrained facilities, routine efficiency may be prioritised over reflective practice, leaving little space for ATs to formalise observations, document welfare-related patterns, or contribute to broader 3Rs discussions. These barriers may be reinforced when ATs have limited access to decision-making forums, when their observations remain informal or localised, and when there are no clear mechanisms to translate practical insights into institutional learning. Limited access to professional development opportunities in communication, data interpretation, or scientific writing may further reduce ATs’ ability to present their expertise in formats that are visible to researchers, managers, or committees. Without institutional recognition and structural support, ATs’ scientific and welfare-related contributions may remain underused, reducing opportunities to identify refinements, improve animal welfare, and strengthen research quality.

This has downstream consequences not only for staff wellbeing but also for experimental consistency, data quality, and the timely implementation of refinements. Evidence from staff wellbeing and Culture of Care scholarship demonstrates that such constraints can negatively affect both personnel wellbeing and animal welfare outcomes. ^12, 13^

Complementary findings from international surveys and workshops show that early-career researchers and technical staff involved in animal research frequently experience structural and communicative barriers that limit engagement, confidence, and participation in experimental decision-making. ^14^ These findings indicate that ATs encounter challenges similar to those faced by early-career researchers, revealing common vulnerabilities. In this context, the educational poster/handout developed as part of this work has relevance beyond ATs and can also support researchers’ professional development. By outlining the essential elements of scientific poster development, the resource provides a practical framework for researchers to strengthen their ability to organise ideas, communicate complex work clearly, and present their contributions with confidence. This reinforces the value of shared communication resources that can support development across roles and career stages within research teams and research facilities.

These observations align with outputs from the IMPROVE COST Action, ^4, 15^ which highlight a widespread desire among animal care staff and technically focused researchers for greater involvement in experimental planning, coupled with a lack of formal mechanisms to support such engagement. The survey included 850 animal care staff and laboratory technicians and identified limited involvement in experimental planning, infrequent access to relevant training and a strong desire for greater participation. Respondents considered that increased involvement could improve communication, job satisfaction, animal welfare and scientific quality. These findings provide broader international support for the need addressed by the “Tell Your Story” workshops, while also showing that communication training should be accompanied by institutional opportunities for ATs to contribute. Further research on this subject would be very well received.

### Building a collective scientific community

These barriers highlight the need to integrate communication and scientific reflection into daily practice, rather than treating them as optional or additional activities. The “Tell Your Story” workshops provide a practical example of how this can be achieved in a low-cost, scalable way within existing institutional settings. However, for such initiatives to have a lasting impact, institutions need to create the conditions that allow ATs to apply these skills in practice.

### Implementation recommendations

In practical terms, this could include giving ATs time within their normal workload to develop and discuss 3Rs ideas, supporting technician-led welfare or refinement projects, offering small internal grants or awards, and encouraging ATs to share their observations through meetings, posters, or institutional events. Institutions could also strengthen ATs’ development by providing access to internal courses on communication, data interpretation, basic statistics, and scientific writing. Embedding these activities into role expectations, appraisal processes, and career-development pathways would help ensure that ATs’ scientific and welfare-related contributions are recognised as part of their professional role, rather than treated as optional work.

### Supporting a shared Culture of Care

Such approaches are consistent with established Culture of Care frameworks and benchmarking initiatives that emphasise shared responsibility, staff engagement, transparency, and reflective practice as foundations for ethical and scientifically robust animal research. ^8, 16, 17^ Educational approaches that explicitly integrate Culture of Care and 3Rs principles into training have also been shown to support shared understanding and professional identity across roles, including technical staff, ^18^ and the present workshop operationalises these principles through a concrete, skills-based intervention focused on communication and confidence-building.

The workshops also highlighted that scientific progress is inherently collaborative and reliant on teamwork. Every member of the research environment, from Animal Technologists to principal investigators, plays a role in maintaining research quality, ethical standards, and animal welfare. Recognising ATs as scientific contributors within this collective framework strengthens institutional resilience and supports a robust Culture of Care.

The workshop, therefore, used poster development as a rehearsal space for scientific dialogue. By preparing and presenting posters, participants practised identifying a key welfare or refinement message, organising evidence, explaining its relevance to research quality, and communicating it to an audience. These are the same core skills required when ATs raise observations with researchers, suggest refinements, contribute to planning discussions, or explain how husbandry and procedural details may affect experimental outcomes. The supportive group format, peer feedback, and opportunity to speak about familiar professional experiences were intended to build confidence, self-efficacy, professional ownership, and constructive assertiveness. These attributes are important foundations for ATs to participate more actively in scientific conversations across hierarchical and disciplinary boundaries.

When ATs adopt scientific reasoning and share their findings, they not only improve animal welfare practices but also shape the broader research culture. Their unique perspective, rooted in daily observation and interaction with animals, complements experimental design and data analysis. Clear communication ensures these insights are shared across roles, strengthening collaboration and supporting ethical, high-quality, and reproducible science.

Taken together, these pilot findings suggest that structured communication interventions, such as “Tell Your Story,” may be relevant beyond a single facility context. By supporting the development of transferable scientific communication skills and encouraging future scientific engagement, the intervention offers a practical model that could be adapted across different animal research environments. However, further research is needed to evaluate its longer-term impact, scalability, and applicability in a wider range of institutional settings.

## Ethics and consent

This paper does not report on a research study in humans. Instead, it includes a reflective account of the “Tell Your Story” workshop activities; as such, no experimental data were collected or analysed. The “Tell Your Story” workshop constituted a knowledge-exchange platform. Participation in the workshop and surveys was voluntary; no personal or indirectly identifiable data were collected, and responses were fully anonymised and reported in aggregate. The fully anonymous electronic survey on non-sensitive topics and the verbal and written (Post-it notes) self-reflection, which demonstrated the workshop benefits (referred to as preliminary survey), plus the service evaluation/performance review (referred to as feedback survey), were considered to present minimal ethical risk in line with UCL Research Ethics guidance. Consequently, as this work does not constitute research involving human participants, formal ethical approval was not required.

Participation in the workshops was voluntary, and attendance indicated willingness to take part. Implied consent was provided through completion of anonymous online surveys. Following the workshops, participants received written information via email outlining the project’s purpose, the type of data collected, and how the findings would be used solely for a manuscript describing the workshop. The email clarified that responses were collected anonymously, would be handled in strict confidence, and presented only in anonymised form. Participants were reminded of their right to decline or withdraw their survey responses at any time without consequence and were offered the opportunity to withdraw after reviewing the results. No participants chose to withdraw from the study.

## Data Availability

Repository Name: [Tell Your Story: Strengthening Scientific Engagement and Welfare Leadership among Animal Technologists]. https://doi.org/10.5522/04/c.8299507. ^5^ This repository contains the following underlying data:
- Statistical Analysis Data: used in Figure 2B-C and 3D; this contains the chi-square test results. Repository Name: [Tell Your Story: Strengthening Scientific Engagement and Welfare Leadership among Animal Technologists]. https://doi.org/10.5522/04/c.8299507. ^5^ This repository contains the following extended data:
- Aggregated Survey Data: used in Figure 2A-C and 3D & E; this shows the number of responses received for each question or statement. - Supplementary Figure 1: Tell your story - poster; this contains a poster on how to create a poster. It can be printed in A3 format to ensure the best resolution. - Supplementary Figure 2: Tell your story - handout; this PDF handout can serve as a helpful guide when designing a poster. It can be printed in A4 format to ensure the best resolution. All materials are available under the terms of the Creative Commons Zero “No rights reserved” data waiver (CC0 1.0 Public domain dedication). **Supplementary Figure 1**. **“Tell your story” poster.** Step-by-step guide (min. A3 size) for creating a poster. **Supplementary Figure 2**. **“Tell your story” handout.** Step-by-step guide (min. A4 size) for creating a poster.
