# Supplementary material for: Tell Your Story: Strengthening Scientific Engagement and Welfare Leadership among Animal Technologists
Source: Wellcome Open Res. 2026 Aug 10;11:243. Originally published 2026 Apr 27. [Version 3] doi: 10.12688/wellcomeopenres.25958.3 (PMC13555090; doi:10.12688/wellcomeopenres.25958.3)
Supplement: Peer review history

# Reviewer report: Reviewer response for version 3

**DOI:** 10.21956/wellcomeopenres.30207.r163915

**Published:** 2026-08-22

**Authors:** David Mawufemor Azilagbetor

**Affiliations:** 1 University of Basel, Basel, Switzerland

I thank the authors for responding to and satisfactorily addressing my comments. I have no further concerns.

Is the work clearly and accurately presented and does it cite the current literature?

Yes

If applicable, is the statistical analysis and its interpretation appropriate?

I cannot comment. A qualified statistician is required.

Are all the source data underlying the results available to ensure full reproducibility?

Yes

Is the study design appropriate and is the work technically sound?

Yes

Are the conclusions drawn adequately supported by the results?

Yes

Are sufficient details of methods and analysis provided to allow replication by others?

Partly

Reviewer Expertise:

Culture of care in animal research; harm-benefit analysis; patient and public involvement

I confirm that I have read this submission and believe that I have an appropriate level of expertise to confirm that it is of an acceptable scientific standard.

# Reviewer report: Reviewer response for version 2

**DOI:** 10.21956/wellcomeopenres.29870.r160344

**Published:** 2026-07-28

**Authors:** Penny Hawkins

**Affiliations:** 1 Royal Society for the Prevention of Cruelty to Animals, Horsham, England, UK

This paper is well-written, addresses an important contemporary issue and provides good, clear advice to help address this. I did feel it could be more succinctly written and there is extraneous detail, e.g. 'to facilitate the workshop, an appropriate venue was selected, and all necessary equipment was arranged prior to the workshop' and the 'purpose/rationale' columns in Tables 1 and 3. The discussion, in particular, included a lot of detail that was not really discussing the workshop outcomes (these are included, but some of the other material is more background than discussion). I would recommend a critical edit to ensure that all the detail is necessary and located in the most appropriate section of the report. A few more detailed comments:

 The para beginning 'then the presenters' is hard to read and needs to be split up into more sentences. There is also repetition in paras 2 and 3 under 'Communication as empowerment and integration'. I also found the graphics in Fig 2 hard to decipher; I personally would have found histograms easier to understand. 

 With respect to referencing; the COST Action is cited but not its output, which needs to be included and discussed (PMID: 41981801). The International Culture of Care Network is not an RSPCA initiative; the best citation for it is [norecopa.no/coc].

Is the work clearly and accurately presented and does it cite the current literature?

Partly

If applicable, is the statistical analysis and its interpretation appropriate?

I cannot comment. A qualified statistician is required.

Are all the source data underlying the results available to ensure full reproducibility?

No source data required

Is the study design appropriate and is the work technically sound?

Yes

Are the conclusions drawn adequately supported by the results?

Yes

Are sufficient details of methods and analysis provided to allow replication by others?

Yes

Reviewer Expertise:

Animal welfare, ethics, regulation of animal care and use

I confirm that I have read this submission and believe that I have an appropriate level of expertise to confirm that it is of an acceptable scientific standard.

# Response

**Published:** 2026-08-01

**Authors:** Eleni Amaniti

**Affiliations:** Sainsbury Wellcome Centre, University College London, London, England, UK

We carefully considered each comment and made targeted changes to improve the manuscript’s clarity and accuracy. These include shortening the venue description, restructuring a complex paragraph, adding the recent COST Action publication, and adding the citation for the International Culture of Care Network.

 We also reviewed the suggestions concerning the Discussion, tables and Figure 2, as detailed below.  

**1. The manuscript could be more succinctly written and contains extraneous detail, for example the description of venue preparation and the purpose/rationale columns in Tables 1 and 3.**

 We have shortened the description of the venue and equipment arrangements. We retained the “Purpose/rationale” columns because they help readers understand how each element contributes to the workshop’s learning objectives and support the manuscript’s aim of providing a reproducible workshop framework.  

**2. The Discussion includes detail that is not directly discussing the workshop outcomes and would benefit from a critical edit.**

 We reviewed the Discussion but retained its current scope. As this is a small pilot workshop, the wider professional and institutional context is important for interpreting the findings and explaining their relevance to professional empowerment, scientific engagement and Culture of Care.  

**3. The paragraph beginning “Then the presenters” is difficult to read and should be divided into more sentences.**

 We have rewritten this paragraph using shorter sentences and a clearer structure.  

**4. There is repetition in paragraphs 2 and 3 under “Communication as empowerment and integration”.**

 We reviewed and clarified these paragraphs.    

**5. The graphics in Figure 2 are difficult to decipher; histograms would be easier to understand.**

 We considered this suggestion but retained the existing format, as Figure 2 provides a compact summary of several survey questions rather than the distribution of continuous data.  

**6. The COST Action is cited, but its output should also be included and discussed (PMID: 41981801).**

 We have added Gonzalez-Uarquin et al. (2026; PMID: 41981801) and acknowledged its relevance to the involvement of animal care staff and laboratory technicians in scientific planning and practice.  

**7. The International Culture of Care Network; the most appropriate citation is norecopa.no/coc.**

 We have replaced the previous citation with the Network’s Norecopa webpage ( https://norecopa.no/coc). We thank the reviewer for this correction.   We thank the reviewer for their thoughtful feedback, which has helped improve the manuscript’s clarity and accuracy while preserving its practical purpose and scope.

# Reviewer report: Reviewer response for version 2

**DOI:** 10.21956/wellcomeopenres.29870.r160114

**Published:** 2026-07-13

**Authors:** Chenyang Li

**Affiliations:** 1 Chinese Academy of Agricultural Sciences, Beijing, China

The authors have addressed my comments satisfactorily. I have no additional comments.

Is the work clearly and accurately presented and does it cite the current literature?

Yes

If applicable, is the statistical analysis and its interpretation appropriate?

Yes

Are all the source data underlying the results available to ensure full reproducibility?

Yes

Is the study design appropriate and is the work technically sound?

Yes

Are the conclusions drawn adequately supported by the results?

Yes

Are sufficient details of methods and analysis provided to allow replication by others?

Yes

Reviewer Expertise:

Animal welfare and behaviour

I confirm that I have read this submission and believe that I have an appropriate level of expertise to confirm that it is of an acceptable scientific standard.

# Reviewer report: Reviewer response for version 1

**DOI:** 10.21956/wellcomeopenres.28570.r154367

**Published:** 2026-06-18

**Authors:** David Mawufemor Azilagbetor

**Affiliations:** 1 University of Basel, Basel, Switzerland

**Summary**

 I believe this work makes a significant contribution to the field. Animal Technologists (ATs) play an instrumental role in the successful conduct of animal experiments. It is understood that healthy, well-cared-for animals make better science. In this respect, the contribution of ATs to animal welfare is indispensable for ensuring the scientific quality of experimental procedures and the validity of the results obtained.

 Below are some issues I think should be addressed.

**Overall aim and output of paper**

***A resource as an output***

 The study is generally described (and it is summarized in the abstract as such) as involving “ *the development of a practical resource designed to support ATs’ self-awareness, confidence, and engagement in scientific discussions by helping them articulate their expertise clearly and effectively. The resources were evaluated during “Tell Your Story” workshops*.” The authors mention that “ *an educational **poster/handout** detailing the essential elements of scientific poster development was created and provided as a resource*”. The authors could consider reflecting and highlighting how this resource, as an output of this research, is applicable within research teams and research facilities.

***Methodological approach as an output***

 Another observable aspect of the paper that should be highlighted is its elucidation of the challenges and opportunities or avenues for ATs to be involved in scientific discussions. The methods used could be described as tools for further research on this subject and, largely, tools for improving ATs’ confidence and engagement in scientific discussions. What could be mentioned about these activities is that they aided the participants to develop skills and obtain encouragement for future scientific engagements.

**How do ATs develop technical expertise**

 The paper is based on scientific communication such as poster design and presentation. The authors should also consider reflecting and briefly discussing in the introduction or discussion that ATs possess the technical or scientific skills, mentioning how and through what channels ATs develop technical expertise (scientific knowledge about animal experiments and the 3Rs), which they are to contribute to scientific discussions. Encouraging and training ATs to contribute to scientific aspects of research is necessary, but before that, they need to have the expertise. The authors can briefly mention this aspect and how ATs obtain it.

**Participants**

 The authors should consider reporting the total number of research institutions (or the number of animal facility departments) that participated in the workshops. It would also be informative to have some information about the overall profiles of the participants from each institution or facility department.

**Recruitment**

 The authors should provide information about how the animal research facility departments expressed interest in the study, particularly the medium through which this took place. Not everyone could participate; " *the final group selected to reflect a mix of roles, career stages, and experience levels*". Did the authors follow any pre-defined selection criteria for this process? How exactly were the participants selected based on these criteria? How was selection bias managed?

**Outcomes of the workshops relative to the objectives of the paper:**

 How do the presentations and communications of the participants during the workshop relate to the main objective of the paper, which is about how ATs can be involved in communication with scientists about experiments? Beyond the poster design, how did the workshops encourage or promote courage (and other attributes) needed for ATs to communicate with scientists?

**Institutional implementation: **Finally, more reflection is needed on how institutions can practically create space for the engagement of ATs in research discussions, putting the contributions of this paper into practice.

Is the work clearly and accurately presented and does it cite the current literature?

Yes

If applicable, is the statistical analysis and its interpretation appropriate?

I cannot comment. A qualified statistician is required.

Are all the source data underlying the results available to ensure full reproducibility?

Yes

Is the study design appropriate and is the work technically sound?

Yes

Are the conclusions drawn adequately supported by the results?

Yes

Are sufficient details of methods and analysis provided to allow replication by others?

Partly

Reviewer Expertise:

Culture of care in animal research; harm-benefit analysis; patient and public involvement

I confirm that I have read this submission and believe that I have an appropriate level of expertise to confirm that it is of an acceptable scientific standard, however I have significant reservations, as outlined above.

# Response

**Published:** 2026-06-25

**Authors:** Eleni Amaniti

**Affiliations:** Sainsbury Wellcome Centre, University College London, London, England, UK

We thank the reviewer for their thoughtful and constructive comments.  

 We are pleased that the reviewer recognised the significant contribution of this work and the indispensable role of Animal Technologists (ATs) in supporting animal welfare, scientific quality, and the validity of experimental outcomes.  We have addressed each point below.

**1. Clarifying the practical resource as an output of the research**

 We agree with the reviewer that the practical resource should be highlighted more clearly as a study output.  We have revised the Discussion to clarify that the educational poster/handout was not intended solely as a guide to poster design, but as a practical communication resource to help ATs structure, articulate, and share their expertise with scientists, managers, Named Persons, and wider research teams. We have also expanded the Discussion to explain how this resource may be used within research facilities, for example, in technician-led welfare or 3Rs projects, internal facility meetings, training sessions, poster events, appraisal discussions, and conversations with researchers about experimental planning, animal welfare, and opportunities for refinement.

**2. Highlighting the methodological approach as an output**

 We agree that the workshop methodology itself represents an important output of the paper.  We have revised the Discussion to make it clearer that the workshop format provides a transferable approach that other institutions could adapt to explore ATs’ experiences, barriers, motivations, and opportunities for scientific engagement.

**3. Explaining how ATs develop technical and scientific expertise**

 We agree that the manuscript should more clearly acknowledge how ATs develop the expertise that enables them to contribute meaningfully to scientific discussions.  We have revised the Introduction and Discussion to emphasise that ATs develop technical and scientific expertise through formal training in animal technology and laboratory animal science, competency-based institutional training, continuing professional development, regulated training requirements, practical experience, and daily observation of animal health, behaviour, welfare, and experimental outcomes.

**4. Reporting the number of participating institutions and participant profiles** We agree that this information improves transparency.  We updated the Methods section to specify that participants were from four institutions, consistent with the Workshop Overview section.  We also want to clarify that, due to the small number of participants and the need to preserve anonymity, participant profiles are reported using broad, aggregated role and experience categories rather than being broken down by individual institution or facility department. These aggregated participant characteristics are shown in Figure 2, which presents the overall distribution of roles and experience levels across the workshops.

**5. Clarifying recruitment, selection, and potential selection bias**

 We agree that the recruitment process requires further clarification.  We have revised the Methods section to clarify that these were pilot workshops and that the institutions approached were selected in advance by the authors to ensure the workshops were manageable and feasible within the available time, staffing, and logistical constraints. We have clarified that an invitation outlining the workshop’s aims and structure was circulated via email to senior management in animal facility departments at institutions that had previously expressed interest in research delivery.   We have also added that, as this was a pilot and exploratory study, the recruitment strategy was not intended to generate a statistically representative sample.  We acknowledge that selection bias may have been present, particularly because institutions and individuals with an existing interest in AT engagement were more likely to participate.  However, we sought to manage this pragmatically by including participants from different institutions, roles, and career stages.

**6. Linking workshop activities to the main objective of the paper**

 We agree that the relationship between the workshop activities and the paper’s main objective needs to be made more explicit.  We have revised the Results and Discussion to clarify that poster design was used as a practical vehicle for scientific communication, rather than being the sole focus of the workshops.

**7. Explaining how the workshops supported confidence, courage, and communication with scientists**

 We agree with the reviewer that this point should be developed further.  We have revised the manuscript (Activities 2/3 and Discussion) to explain that the workshops aimed to create a supportive environment in which ATs could reflect on their own scientific role, share experiences with peers, and practise communicating their expertise in a structured and constructive way. We now describe the workshops as supporting confidence, self-awareness, professional ownership, peer support, and constructive assertiveness.  

**8. Institutional implementation and creating space for AT engagement**

 We agree that the manuscript would benefit from more practical reflection on how institutions can create space for ATs to engage in research discussions.  We have expanded the Discussion to emphasise that communication and scientific reflection should be embedded into routine facility and research culture, rather than treated as optional or additional activities.   We hope these revisions have strengthened the manuscript's clarity, methodological framing, and practical applicability, particularly regarding how the resource and workshop approach can support AT engagement in scientific communication within research facilities and teams.

# Reviewer report: Reviewer response for version 1

**DOI:** 10.21956/wellcomeopenres.28570.r154366

**Published:** 2026-05-11

**Authors:** Chenyang Li

**Affiliations:** 1 Chinese Academy of Agricultural Sciences, Beijing, China

The study design is appropriate, and the manuscript is generally well structured and clearly written. However, several aspects could be further strengthened to improve the academic rigour of the paper.

 In the introduction section, the authors could further emphasise how the under-recognition of ATs’ scientific contributions may negatively affect the implementation of the 3Rs principles and the improvement of animal welfare. Expanding on these implications would better highlight the urgency and significance of the study.

 In the “Location requirements” section, the abbreviation “SWC” appears without prior explanation. I assume this refers to the Sainsbury Wellcome Centre, but this can only be inferred after checking the authors’ affiliations. It is recommended that the full name be provided when “SWC” first appears in the manuscript.

 For Figure 1, it is recommended that the “Learning Outcomes” be briefly explained in the figure legend, or alternatively, replaced with the same wording used on Page 8.

 In Figure 2A, the colours used for different categories are too similar (e.g., “Senior AT” and “Manager”), making them difficult to distinguish. It is recommended that the authors use colours with greater contrast or consider replacing the pie chart with a bar chart. Similar improvements could also be considered for the presentation of other results in Figure 2.

 The manuscript applies a chi-square goodness-of-fit test; however, the theoretical rationale for the assumed expected distribution (i.e., a uniform distribution across Likert scale categories) is not clearly stated. The authors should provide a justification for this assumption or clarify the underlying statistical rationale.

 The study includes a relatively small sample size (n = 27) from four institutions. Although the sample provides some degree of representativeness, the statistical inference remains limited. It is recommended that the authors more explicitly describe the study as a pilot study in both the abstract and discussion sections and avoid overgeneralising the conclusions.

 In the section “Communication as empowerment and integration”, the manuscript highlights the role of communication training in promoting cultural integration; however, institutional-level barriers (e.g., management structures, resource allocation, and incentive mechanisms) are not sufficiently discussed. The authors are encouraged to provide more concrete suggestions regarding how institutions could facilitate the adoption of such initiatives.

 In the section “Building a collective scientific community”, the manuscript describes the resource as a “low-cost, scalable manner”; however, no specific estimation of costs (e.g., materials, time, or personnel) or strategies for implementation in other institutions are provided. The authors may consider adding a brief “Implementation Recommendations” subsection to improve the practical applicability of the study.

Is the work clearly and accurately presented and does it cite the current literature?

Yes

If applicable, is the statistical analysis and its interpretation appropriate?

Yes

Are all the source data underlying the results available to ensure full reproducibility?

Yes

Is the study design appropriate and is the work technically sound?

Yes

Are the conclusions drawn adequately supported by the results?

Yes

Are sufficient details of methods and analysis provided to allow replication by others?

Yes

Reviewer Expertise:

Animal welfare and behaviour

I confirm that I have read this submission and believe that I have an appropriate level of expertise to confirm that it is of an acceptable scientific standard, however I have significant reservations, as outlined above.

# Response

**Published:** 2026-06-25

**Authors:** Eleni Amaniti

**Affiliations:** Sainsbury Wellcome Centre, University College London, London, England, UK

We appreciate the reviewer's thoughtful and constructive feedback.  We're glad to hear that the study design was suitable and that the manuscript was well organised and clear.  We have responded to each comment below.

**1.  Emphasising the implications of under-recognising Animal Technologists’ scientific contributions**

 We agree with the reviewer that this point could be made more explicit.  We have strengthened the Introduction to further emphasise how under-recognition of ATs’ scientific contributions may limit the implementation of the 3Rs, reduce opportunities for welfare refinement, and weaken communication between technical and research teams.

**2.  Definition of SWC**

 We thank the reviewer for identifying this.  We have now written out “Sainsbury Wellcome Centre” in full at first mention, followed by the abbreviation “SWC”.

**3.  Figure 1: clarification of Learning Outcomes**

 We agree that this requires clarification.  We have updated the wording on what we believe is page 8 to match the figure, ensuring consistency with the main text.

**4.  Figure 2: colour contrast and presentation of results**

 We thank the reviewer for this helpful suggestion.  We have revised the presentation of Figure 2 to improve readability, including the use of higher-contrast colours.

**5.  Statistical rationale for the chi-square goodness-of-fit test**

 We agree that the rationale for the expected distribution needed to be clearer.  We have revised the Methods (Software & Statistical analysis section) and Figure legend to explain the rationale for using a uniform expected distribution across Likert categories, and to acknowledge the limitations of this assumption in the context of a small pilot study.

**6.  Small sample size and pilot study framing**

 We agree with the reviewer.  We have revised the Abstract and Discussion to more explicitly describe the work as a pilot study.  We have also softened the conclusions to avoid overgeneralisation and to emphasise that the findings should be interpreted as preliminary and hypothesis-generating.

**7.  Institutional-level barriers and practical adoption**

 We recognise that institutional barriers are significant challenges.  We have expanded the Discussion to explicitly refer to barriers such as management structures, workload pressures, resource allocation, and recognition mechanisms.  We have also added more concrete suggestions for how institutions could support adoption, including protected time, technician-led welfare or refinement projects, internal presentation opportunities, and access to relevant training.

**8.  Low-cost and scalable implementation**

 We thank the reviewer for this suggestion.  We have added further detail on implementation, including an indicative average material cost for the workshop resource packs.  We have also added an “Implementation recommendations” subsection to clarify how similar initiatives could be adopted in other institutions, including through modest structural changes such as setting aside time for reflection, supporting poster development, and embedding AT contributions within appraisal and career-development pathways. We hope these revisions have strengthened the manuscript's academic rigour, clarity, and practical applicability.
